# Assessing exposure from different vehicular antennas in military applications: a computational study

**DOI:** 10.3389/fpubh.2025.1620240

**Published:** 2025-08-26

**Authors:** Micol Colella, Marianna Biscarini, Daniele Ferrante, Giovanni Pellegrino, Marco De Meis, Luca Mei, Marta Cavagnaro, Francesca Apollonio, Micaela Liberti

**Affiliations:** ^1^Department of Information Engineering, Electronics and Telecommunications, Sapienza University of Rome, Rome, Italy; ^2^Centro Polifunzionale di Sperimentazione (CEPOLISPE), Rome, Italy; ^3^Larimart S.p.A., Rome, Italy

**Keywords:** occupational exposure assessment, computational dosimetry, military personnel, radiating antenna, near field exposure

## Abstract

**Introduction:**

Military personnel frequently operate in close proximity to electromagnetic (EM) sources such as vehicular communication antennas. Despite this occurrence, detailed evaluations of exposure scenarios remain limited. This study bridges this gap by examining EM exposure from military vehicular antennas, covering a broad spectrum of frequencies (high frequency—HF, very high frequency—VHF, ultrahigh frequency—UHF), power levels, and positions.

**Methods:**

The study used computational modeling to simulate realistic military scenarios, including personnel partially outside armored vehicles and equipped with personal protective equipment. Simulations accounted for a broad spectrum of frequencies (HF, VHF, and UHF) as well as different power levels, antenna types and locations.

**Results:**

The analysis revealed substantial variability in EM exposure levels depending on the configuration and conditions. While all simulated scenarios complied with the ICNIRP Basic Restrictions (BR), certain cases exceeded the Reference Levels (RL), particularly under specific positioning and frequency combinations.

**Conclusions:**

These findings, based on a detailed case-specific analysis, suggest that personnel safety in military contexts is generally maintained, even in the presence of variable exposure conditions and elevated levels of radiated E-field. Considering the basic structure of the radiating source (i.e., monopole) and the nature of near-field interactions, this paper suggests that safe exposure conditions could be expected to persist across a range of antenna-operator positioning configurations, and building on previous preliminary research on this topic, it provides relevant insights for operational instructions and improving safety regulations in the military field.

## 1 Introduction

Electromagnetic (EM)-based technologies are becoming increasingly prevalent, influencing various aspects of daily life and work. Consequently, there is a growing need to understand how EM sources interact with the human body and to develop methods that ensure compliance with safety guidelines (1–3). Defense is one of the sectors that heavily depend on EM-based technologies, where EM-fields are primarily used for communication purposes, under conditions substantially different from those applied in civilian applications (4, 5). Operational waveforms, frequencies and power levels are governed by standardized frameworks (e.g., MIL-STD and STANAG), which aim to optimize functionality and interoperability (6), e.g., this applies among NATO members and strategic partners. As a result, military systems often share similar technical characteristics across nations. Military vehicles are equipped with antennas operating across a broad frequency range, including high frequency (HF), very high frequency (VHF), and ultra-high frequency (UHF). These antennas emit high power levels required for communication and jamming and are often placed in proximity to crew-access points, such as manholes, resulting in personnel exposure conditions that fall within the near field regime (7). Despite the peculiar conditions, regulatory approaches in this context remain highly fragmented and largely dependent on national policies. A military-specific standard was introduced in 2014 by IEEE TC95 (8) to replace the NATO Standardization Agreement (STANAG) (9), but it is currently listed as Inactive-Reserved, as it undergoes its 10-year revision cycle. In contrast, the International Commission on Non-Ionizing Radiation Protection (ICNIRP) (10), which serves as the scientific basis for the European Directive 2013/35/EU, classifies military scenarios under the occupational exposure category, applying the limits without specific adaptations for military environments. ICNIRP limits are defined in two primary ways: (i) reference levels (RL), which represent unperturbed electric and magnetic fields intensity or power density values in areas accessible to humans; (ii) basic restrictions (BR), which are EM quantities induced inside the human body and directly related to observed health effects at specific frequencies. Compliance is typically demonstrated using RL; however, BR assessments are required when RL are exceeded. A recent review highlighted the diversity of military EM sources, and differences in their distances to personnel, in working frequencies, and power levels (11). Indeed, some exposure conditions have been investigated through measurement campaigns, while others have been evaluated numerically. However, results reveal high variability, including scenarios of non-compliance with safety guidelines (5, 12–14). Computational dosimetry plays a crucial role in assessing exposure, as it enables the calculation of induced EM quantities inside the human body, that would be inaccessible through direct measurements. This is especially relevant when measurements indicate overexposure according to ICNIRP RL, as it allows to verify compliance with BR. Several works showing computational exposure assessment in civilian applications have been carried out over the last decades (15–22), while there is a lack in the military field, due to limited information about specific radiating sources and conditions that prevent accurate modeling. Consequently, most numerical studies on this topic rely on simplified models, generic radiating sources, or homogeneous human phantoms, which do not fully capture the complexities of military scenarios (23–27). To address this gap, recent research by the authors has focused on investigating realistic scenarios that reflect actual working conditions for military personnel. In the first of these studies (28), an operator partially outside a vehicle's manhole was modeled to assess exposure to a 16 MHz vehicular antenna. The analysis allowed to investigate the influence of personal protective equipment (PPE), often overlooked in similar evaluations. We found that while exposure at 25 W power level complied with BR, a local hotspot was detected in the ears due to the cabled headset of the PPE. Successive studies extended this initial work. First, the same 16 MHz antenna was studied with a more realistic operator posture and positioning, i.e., with the operator leaning towards the manhole edge with one arm resting on the vehicle external surface, both with and without PPE (29), to understand the PPE effect on the induced SAR. Then, considering this same realistic conditions, another commonly used antenna operating at 35.5 MHz and 85.5 MHz with 50 W input power was investigated (30). Results indicated potential RL exceedance in all cases, though dosimetric analysis confirmed BR compliance. At 85.5 MHz, hotspots were identified at the knees and ankles, due to E-field penetrating the vehicle's interior. These preliminary studies highlighted the importance of using realistic models for accurate evaluation in operational conditions. Building on these earlier results, in the present work the investigation involves additional frequencies in the HF and VHF bands, and additional antennas in the UHF band, with different power levels and locations on the vehicle. To the authors' knowledge, this study represents the first in-depth exposure analysis in the military setting. By incorporating a realistic operator posture and personal protective equipment (PPE), the study evaluates how EM fields radiated by monopolar antennas for military applications interact with the human body, and how exposure is influenced by frequency, transmitted power, and antenna configuration. Results show that, despite radiated E-field values exceeding ICNIRP reference levels, the exposure remains compliant with basic restrictions due to the absorption from the body. Given the recurring nature of source types and configurations in military environments, these results can be extended to similar scenarios, providing supportive evidence for the general safety in similar exposure conditions and contributing to the formulation of operational guidance and of regulatory frameworks specific to military contexts.

## 2 Methods

### 2.1 Vehicular antennas and exposure scenarios

**Figure 1:**
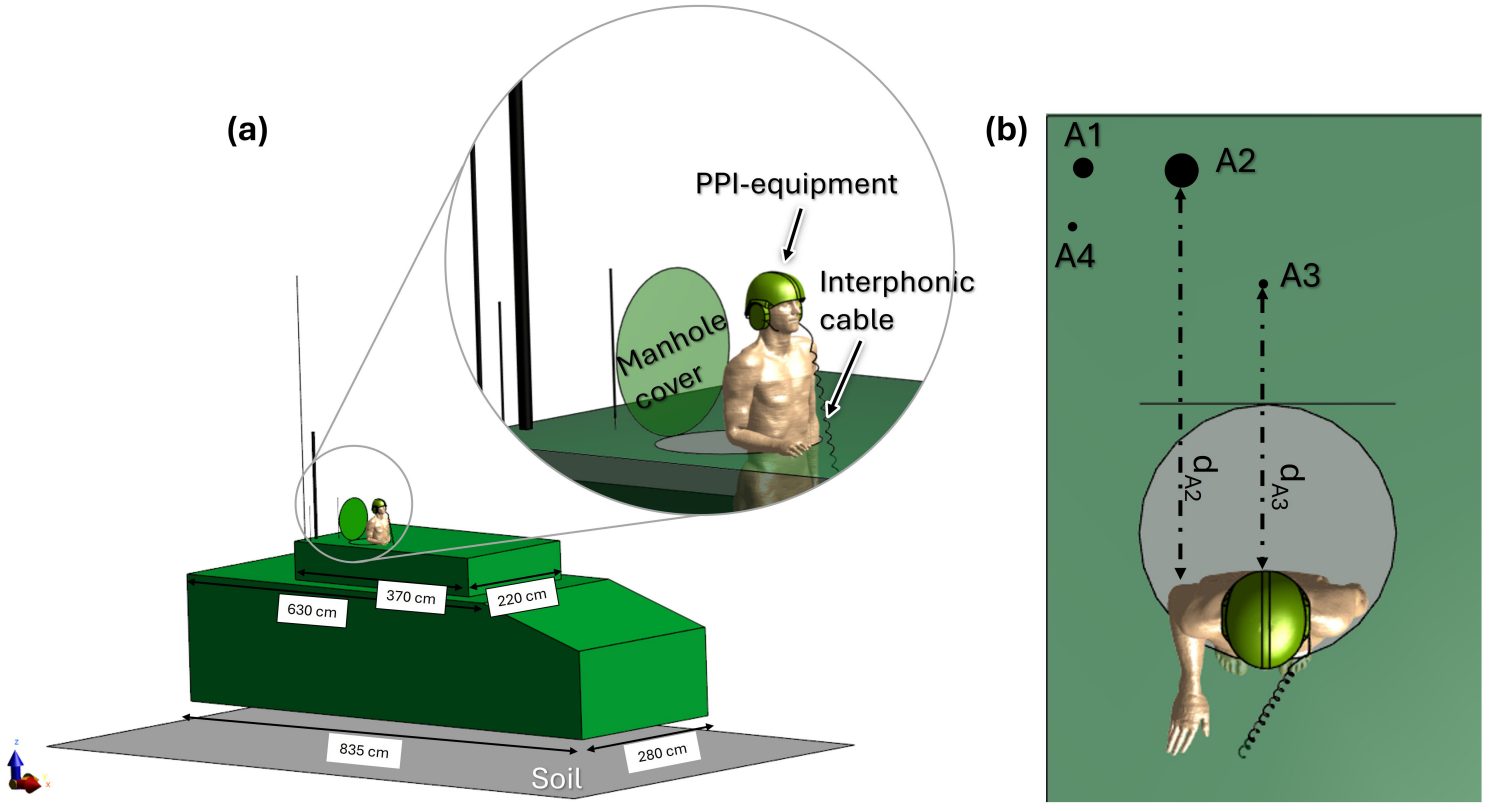
Numerical model of the exposure scenario. **(a)** Prospective view: vehicle model with its dimensions labeled, showing the military operator partially emerging from the manhole and wearing PPE. All simulated antennas are displayed in their mounted positions on the vehicle. The inset provides a detailed view of the operator's posture and PPE configuration. **(b)** Top view: vehicle model from above, with each antenna labeled. Antenna working frequencies are as follows: Antenna 1: 16 MHz, 25 MHz; Antenna 2: 35.5, 60.5, and 85.5 MHz; Antenna 3: 310 MHz; Antenna 4: 351 MHz. Dashed arrows indicate distance from the body for Antenna 2 (d_A2_ = 110 cm) and Antenna 3 (d_A3_ = 74 cm).

The vehicle model used in this study is a simplified 3D numerical representation of a real military tank, consistent with prior research (28–30). Geometry and main dimensions are shown in Figure 1a. A circular aperture in the turret represents the open manhole, through which the operator's body can partially emerge. Four different antennas were evaluated, positioned on the turret in locations corresponding to their actual placements on the tank, as depicted in Figure 1b. Following the methodology applied in previous studies (28–30), each antenna was modeled as a monopole with a height close to λ/4 (where the wavelength λ is determined by the central frequency of the corresponding working band). The diameter and the feeding gap of each antenna were selected according to standard antenna theory (31). Operational conditions, in terms of frequency and maximum operating power (P_op, max_), were provided by the CEPOLISPE (Polifunctional experimental center of the Italian Army, Montelibretti, Rome, Italy) and reflected typical use cases, as described below:

- **Antenna A1:** This HF antenna operates within the 2–30 MHz frequency range. It is 4.20 m high, with a diameter of 2 cm, a 5 cm feeding gap, and it is fed at P_op, max_ of 25 W. In (28) it was studied at 16 MHz; in this study the frequency of 25 MHz is added to the assessment.
- **Antenna A2:** This VHF antenna, studied in (30), is 1.7 m high with a diameter of 6 cm and a 2 cm feeding gap. It operates in the 30–88 MHz frequency range and is fed at its P_op, max_ of 50 W. Here, conditions at 60.5 MHz are assessed alongside earlier evaluations.
- **Antenna A3 and A4:** These UHF antennas are modeled as follows:

° Antenna A3 is 60 cm high, with a diameter of 1 cm and a 5 cm feeding gap operating in the 292–318 MHz frequency range. It is evaluated at 310 MHz at its P_op, max_ of 50 W.
° Antenna A4 is 22 cm high, with a diameter of 1 cm and a 2 cm feeding gap operating in the 225–512 MHz frequency range. It is evaluated at 351 MHz at its P_op, max_ of 20 W.

Each antenna was analyzed independently. First, the Electric field (E-field) generated was studied without the presence of the human model to verify compliance with ICNIRP RL (10). Subsequently, operator exposure was investigated using the whole-body male model Duke [Virtual Population, ViP v.3.1 (32)] representing a 34-year-old man, 1.77 m tall and weighing 70 kg. The model was positioned with 70 cm of the torso protruding through the manhole, leaning toward its edge with one arm bent and resting on the turret surface, as illustrated in the inset of Figure 1a. The PPE, including a helmet and cabled headset, was included in the simulation. The helmet model consists of a ballistic shell and protective foam, the headset includes an intercom cable, consistent with the configurations used in previous studies (28–30).

### 2.2 Simulations set-up

The simulations were performed using Sim4Life (v.7.2, Zurich MedTech AG, Zurich) with the finite difference time domain (FDTD) solver. For each antenna, a non-uniform grid was used to discretize the simulation domain. The vehicle was discretized with a maximum step size of 5 cm along the three orthogonal directions while for the manhole cover a step size of 4 cm was used. Duke's body and the helmet were discretized with an isotropic grid of 2 mm resolution. The cable's wire and teflon jacket employed adaptive subgridding, with a step size of 0.9 mm in the three orthogonal directions. For the antennas, the grid resolution was determined by their geometric dimensions, with a maximum step of 5 mm along the *X* and *Y* axes and 2 cm along the *Z* axis. Antennas and the vehicle were modeled as perfectly electric conductors (PEC). The materials for the PPE were Polyimide and Nylon, consistently with previous studies (28–30). Dielectric properties of the human body were assigned according to the IT'IS Database for each specific working frequency (33). The soil was modeled as a PEC plane (9 m × 5 m); a 60 cm gap was considered between the vehicles and the soil to account for the tires, as done in (28).

## 3 Results

### 3.1 E-field distribution in absence of the human body

**Figure 2:**
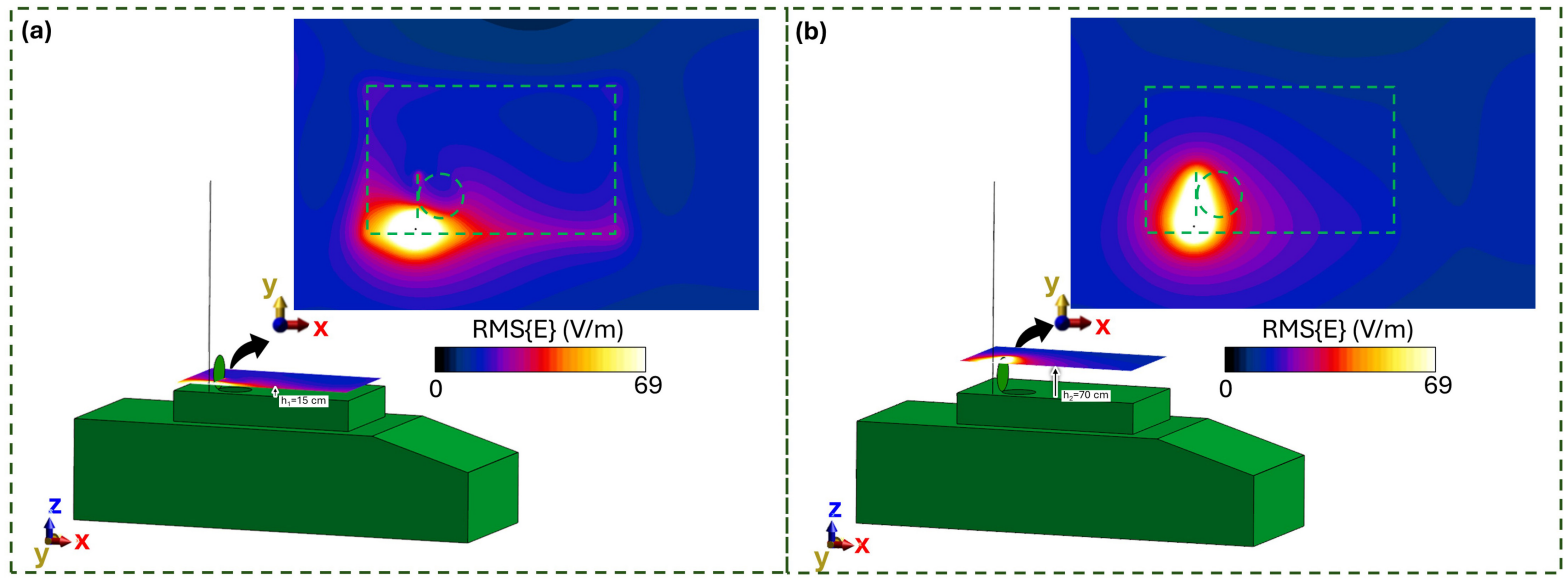
E field generated by Antenna 1, working at 25 MHz and fed with 25 W, in absence of the military operator: **(a)** 15 cm from the manhole; **(b)** 70 cm from the manhole. In dashed green is the hidden profile of the vehicle turret and manhole.

**Figure 3:**
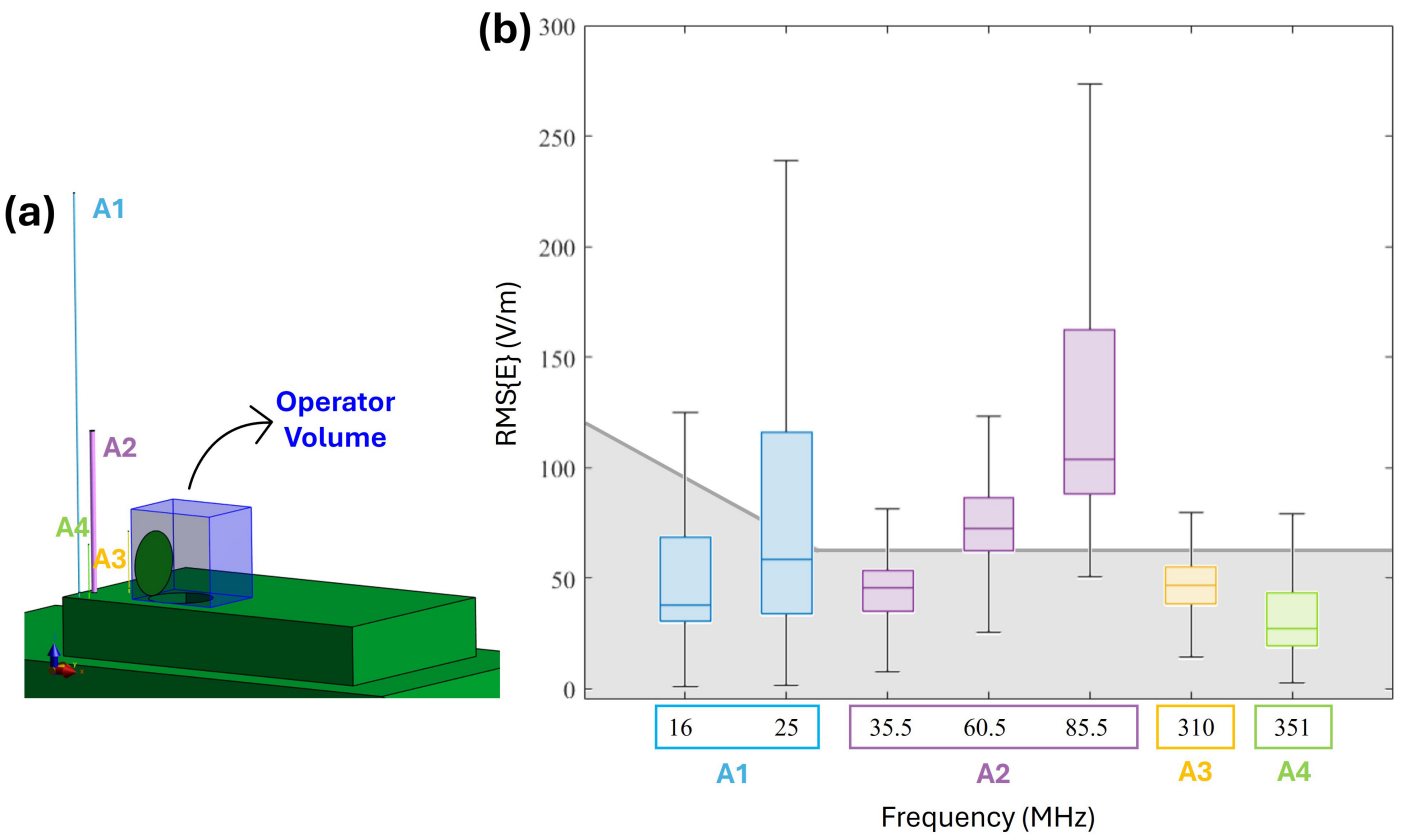
E-field distribution in the *Operator volume* (i.e., free space region occupied by the human body): **(a)** Vehicle model with antennas, the *Operator volume* is highlighted in blue. **(b)** Boxplot of RMS E-field intensities within the *Operator volume*. The shaded gray area represents the ICNIRP 2020 reference levels for the frequency range 10–400 MHz (with the solid gray line indicating the RL threshold).

Figure 2 illustrates the root mean square (RMS) E-field distribution radiated by Antenna 1 at 25 MHz with an input power of 25 W. The results are presented in two planes parallel to the turret surface: one at 15 cm above the manhole (Figure 2a), approximately corresponding to the operator's waist, and another at 70 cm (Figure 2b), roughly at the operator's forehead. The color scale corresponds to the ICNIRP reference level (RL) for 25 MHz [69 V/m (10)], highlighting regions where the E-field reaches or exceeds this threshold. At 15 cm, the E-field distribution is shaped by structural features such as the turret, the manhole, and its cover (indicated by green contours). The regions exceeding 69 V/m are localized around the antenna and partially overlap the manhole area, which might be occupied by the operator. In contrast, at 70 cm, the E-field intensities exceeding 69 V/m are confined within the manhole area, largely influenced by the manhole door. These findings suggest that under these specific conditions, the ICNIRP RL may be exceeded in certain operator regions, which is consistent with prior results at 16 MHz and 25 W (28, 29), as well as at higher frequencies and power levels (30). To assess compliance with ICNIRP RL across the operator's entire potential exposure region, the E field distribution was evaluated within the volume above the manhole, termed the *Operator Volume* (Figure 3a). This volume encompasses all potential positions of the operator's body. Figure 3b summarizes the spatial E-field distribution within this volume as boxplots and compares it to the ICNIRP RL across a frequency range of 16–351 MHz. The ICNIRP RL varies by frequency, with a frequency-dependent value below 30 MHz and a constant value of 61 V/m above 30 MHz. The analysis highlights multiple scenarios of non-compliance with the ICNIRP RL. For Antenna 1, the E-field at 16 MHz and 25 W shows upper whiskers exceeding the RL, while at 25 MHz and 25 W, are the E-field intensities above the median value to exceed 69 V/m. Antenna 2 demonstrates similar trends: at 35.5 MHz and 50 W, the upper whisker exceeds 61 V/m, and at higher frequencies (60.5 and 85.5 MHz, both at 50 W), almost all data points surpass the RL. Antenna 2 produces the highest E fields at these frequencies. For Antenna 3 at 310 MHz and 50 W, and Antenna 4 at 351 MHz and 20 W, the upper whiskers again exceed 61 V/m, indicating RL violations. These findings emphasize the dependence of the radiated E field on antenna type, operating frequency, and input power. In particular, higher frequencies yield greater E-field intensities for the same antenna type (e.g., A1 and A2). As the ICNIRP RL are exceeded in multiple conditions, further computational dosimetry studies incorporating human body models are necessary to evaluate compliance with ICNIRP BR.

### 3.2 Human body exposure

**Figure 4:**
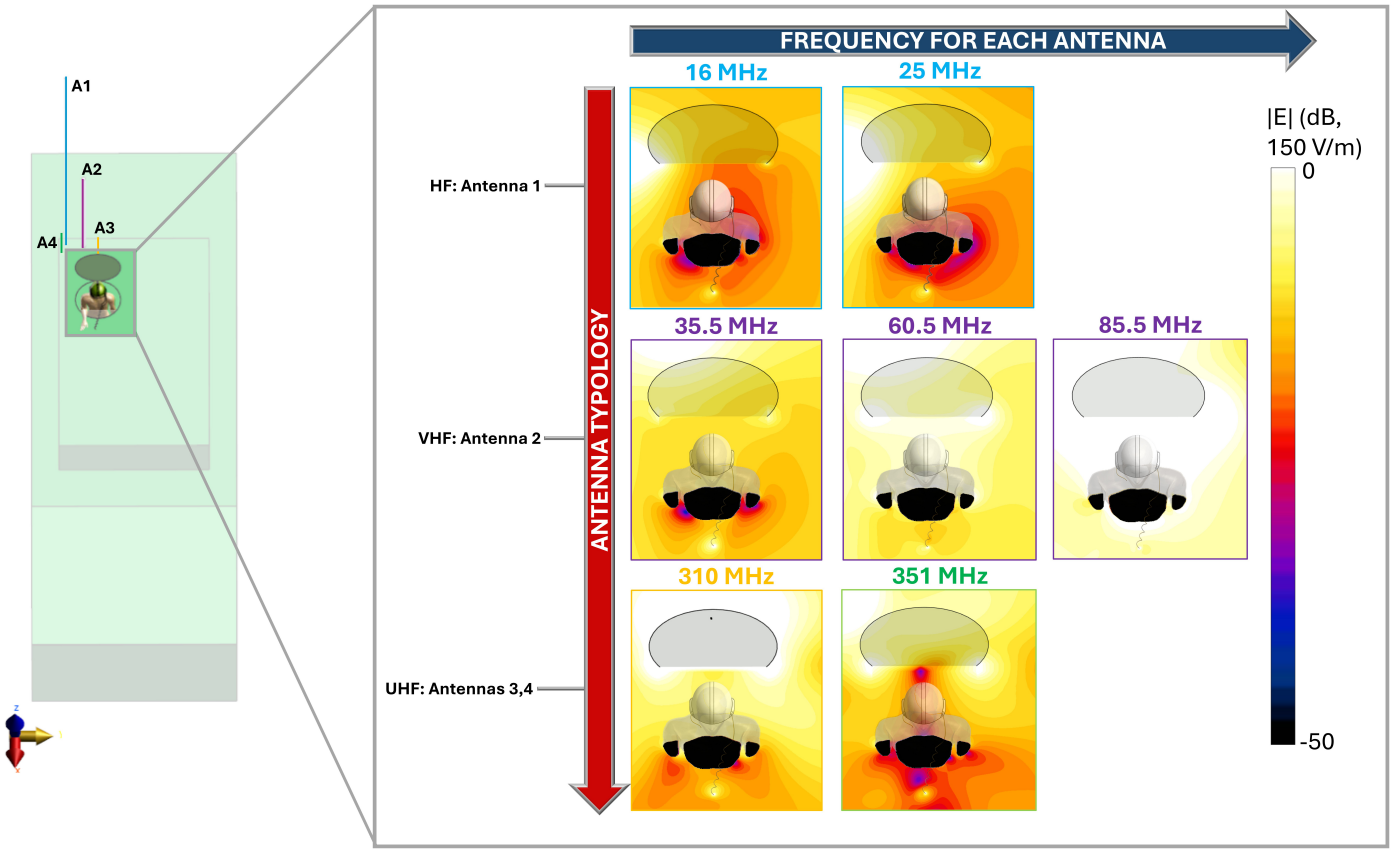
Radiated E-field around the human body. E-field distribution is shown on a XY plane located 15 cm above the manhole surface. Antennas are classified by type (HF—Antenna #1, VHF—Antenna #2, UHF—Antennas 3 and 4) and operating frequency. Each antenna is represented using a color scheme consistent with the coding in Figure 3. The black areas correspond to regions within the operator body where the field is not represented, while in transparency, a portion of the manhole cover appears above the plane of interest.

**Figure 5:**
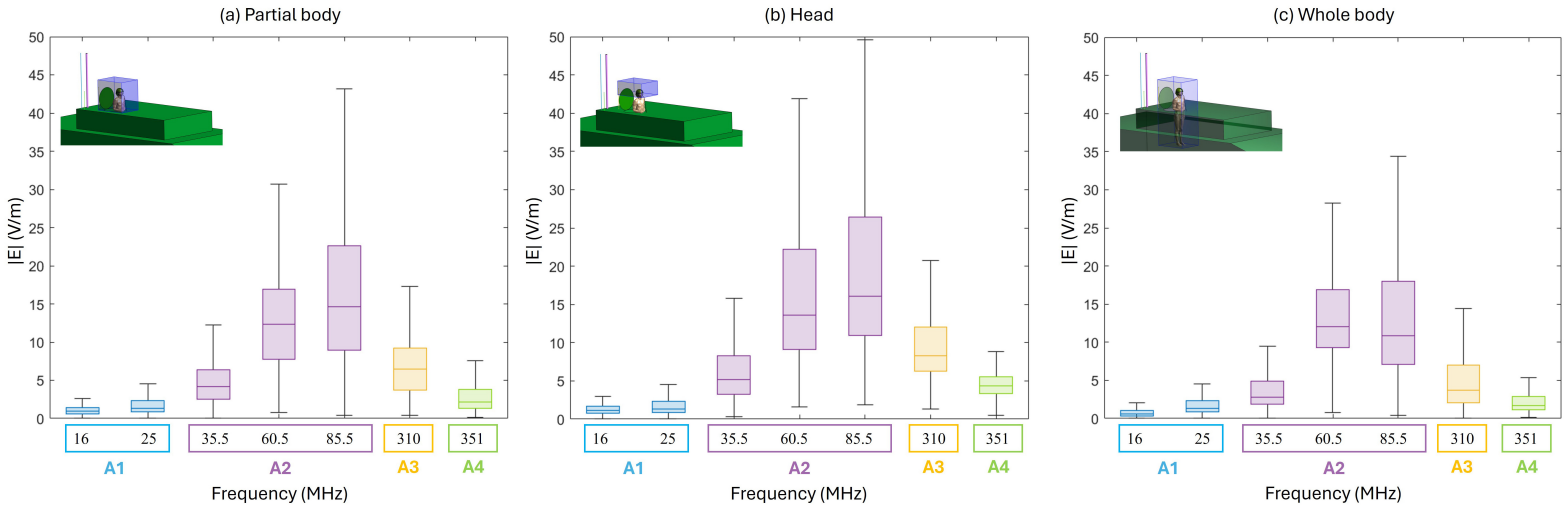
Volumetric distribution of the E-field induced in the body by each Antenna: **(a)** Partial body distribution within the *Operator volume*. **(b)** Head volume distribution. **(c)** Whole-body distribution.

**Figure 6:**
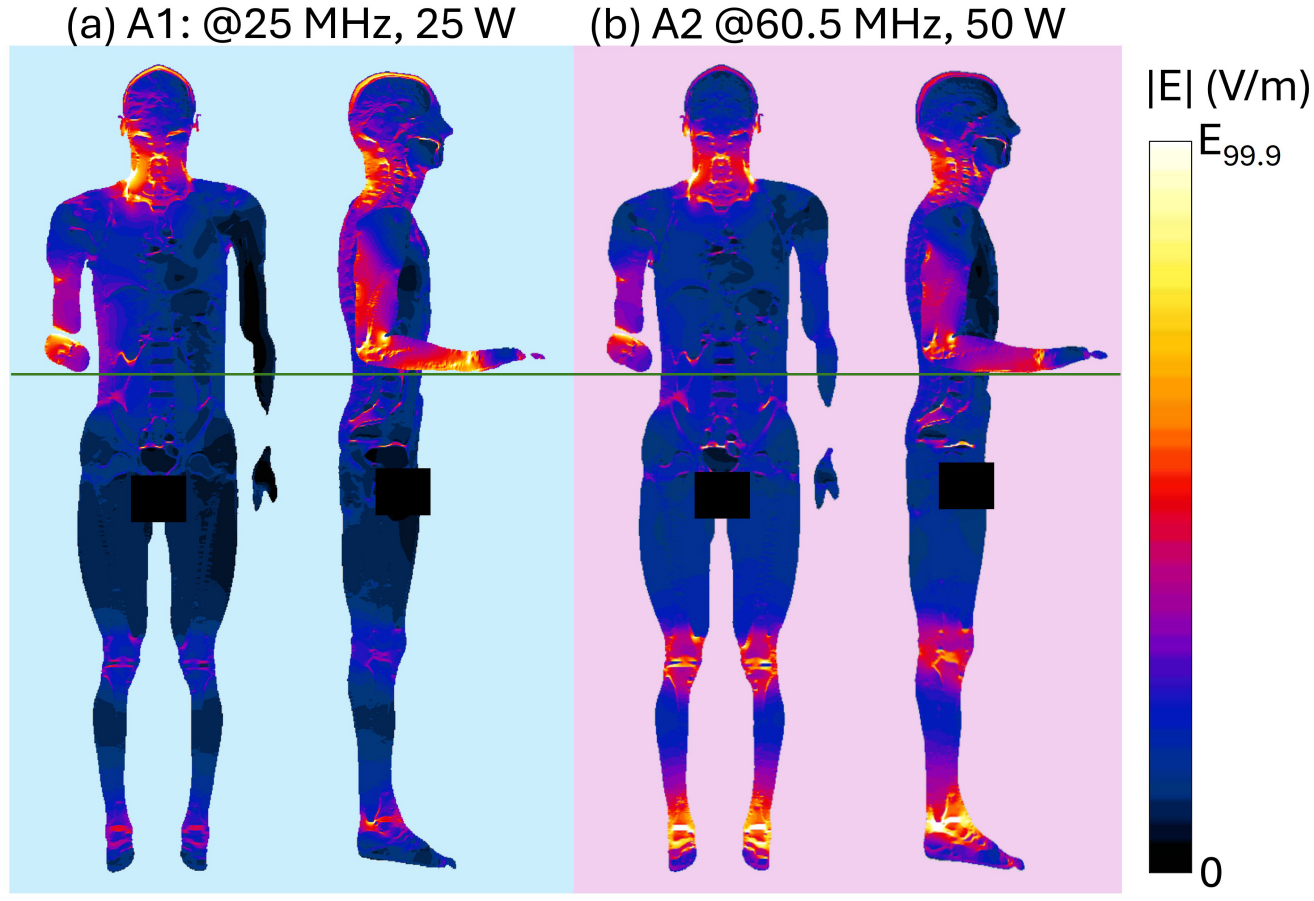
Induced E-field distribution at HF and VHF within planar sections of the human body. Front view shows a section crossing the center of the body. Side view shows three superimposed sections crossing the center of the right arm, of the right leg and of the body: results for **(a)** HF Antenna #1 at 25 MHz, P_in_ = 25 W; **(b)** VHF Antenna #2 at 60.5 MHz, P_in_ = 50 W. Each induced E-field map is shown with a color scale normalized to the 99.9th percentile (E_99.9_) of the whole-body E-field (see Table 1).

**Figure 7:**
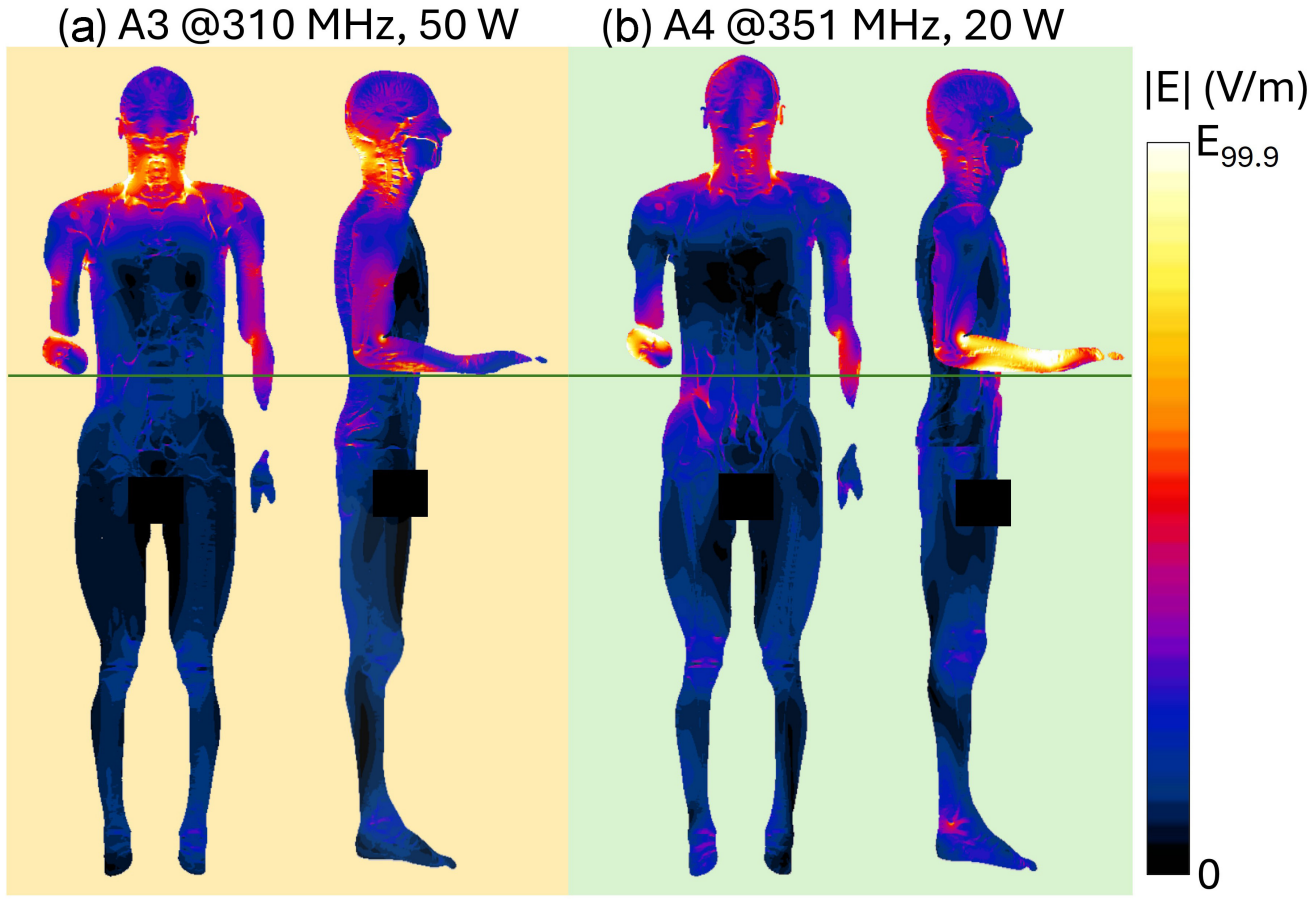
Induced E-field distribution at UHF within planar sections of the human body. Front view shows a section crossing the center of the body. Side view shows three superimposed sections crossing the center of the right arm, of the right leg and of the body: results for **(a)** UHF Antenna #3 at 310 MHz, Pin = 50 W; **(b)** UHF Antenna #4 at 351 MHz, Pin = 20 W. Each induced E-field map is shown with a color scale normalized to the 99.9th percentile (E_99.9_) of the whole-body E-field (See Table 1).

**Figure 8:**
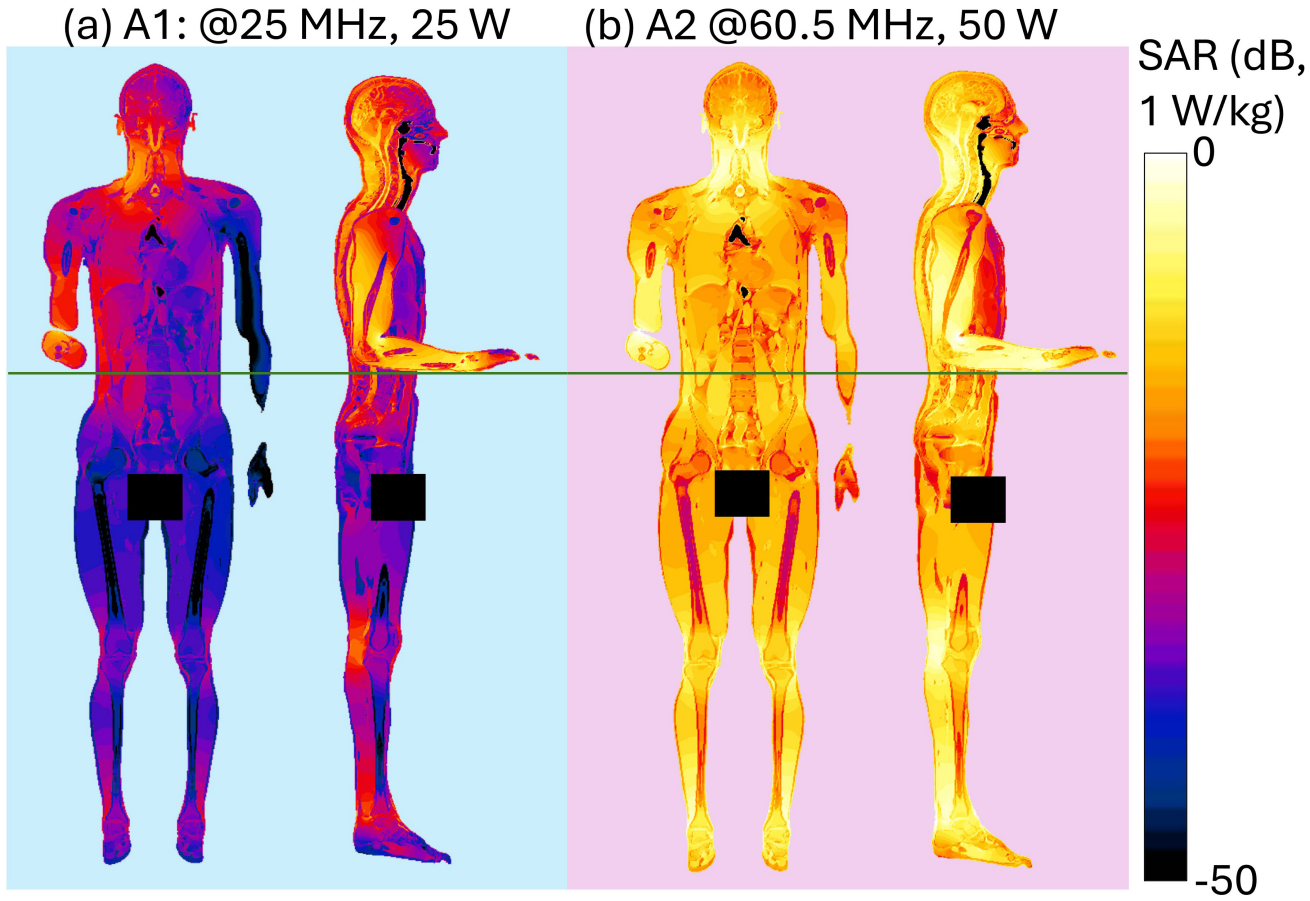
Induced SAR distribution at HF and VHF within planar sections of the human body. Front view shows a section crossing the center of the body. Side view shows three superimposed sections crossing the center of the right arm, of the right leg and of the body: results for **(a)** HF Antenna #1 at 25 MHz, P_in_ = 25 W, **(b)** VHF Antenna #2 at 60.5 MHz, P_in_ = 50 W.

**Figure 9:**
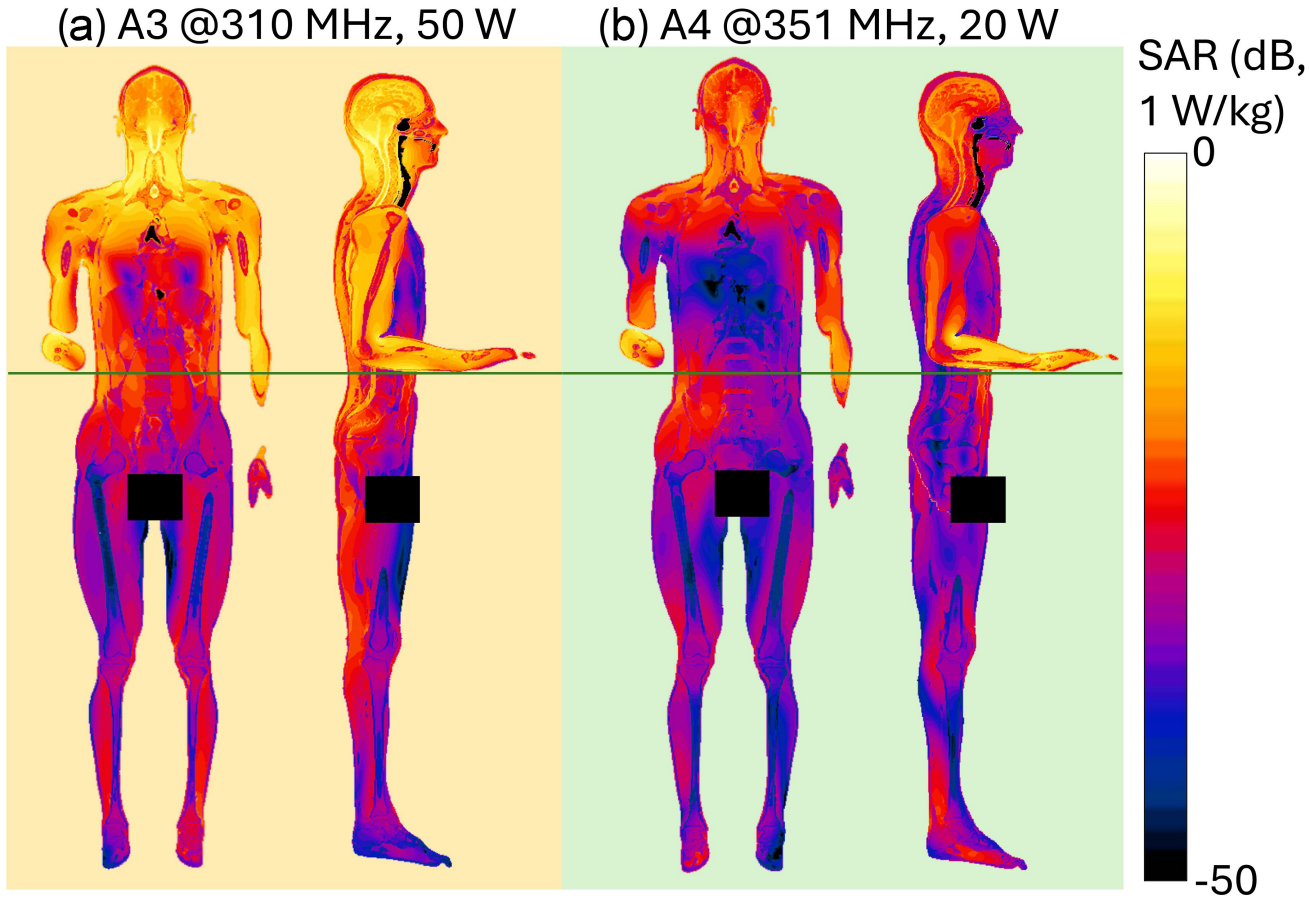
Induced SAR distribution at UHF within planar sections of the human body. Front view shows a section crossing the center of the body. Side view shows three superimposed sections crossing the center of the right arm, of the right leg and of the body: results for **(a)** UHF Antenna #3 at 310 MHz, P_in_ = 50 W, **(b)** UHF Antenna #4 at 351 MHz, P_in_ = 20 W.

Figure 4 shows the spatial distribution of the radiated E-field amplitude in presence of the operator model on planes located at 15 cm above the manhole, parallel to the turret, for each exposure scenario. Field intensity is represented on a dB scale, where 0 dB corresponds to 150 V/m. Note that in the figure, the field distribution inside the body is not represented, and for this reason, the areas within the body are shown in black, emphasizing the radiated field distribution in the external space. Each antenna was fed with the specific P_op, max_ described in Section 2.1. The presence of the operator significantly alters the E-field distribution. At HF (i.e., 16 and 25 MHz), high intensities of the E-field are concentrated near the operator's right arm, with a secondary peak near the left armpit. Conversely, regions around the back and chest experience reductions to 4 V/m (−30 dB). At VHF (35.5, 60.5, and 85.5 MHz), the E-field intensifies near the back and the arms, with peaks reaching 50 V/m (−9 dB), 120 V/m (−2 dB), and 250 V/m (4 dB), respectively. At 35.5 MHz, minima appear around the right ribs and left arm, a pattern not observed at 60.5 MHz or 85.5 MHz. Finally, at UHF, the operator's body shields part of the radiated E-field, reducing intensities in the space directly ahead. Here, in all conditions, localized E-field hotspots are visible and are due to the intercom cable connected to the vehicle. However, the arms determine a concentration of higher E-field values. Despite the high E-field intensities surrounding the body, at the evaluated frequencies the absorption inside the body is extremely low, and the induced E-field is strongly attenuated with respect to the external one. This is shown in Figure 5, where the distribution of the induced E-field inside specific body volumes is reported in boxplots for each antenna and frequency studied. Three different volumes have been investigated: (a) Partial body volume, (b) Head volume, and (c) Whole body volume, all showing similar trends and intensities. The frequency trend resembles the typical human body absorption curve, despite being in near field conditions. Interestingly, also the peak of absorption occurs at similar frequencies: 60.5 and 85.5 MHz, where the median induced E-field is 12 and 15 V/m, respectively, when evaluated in the partial body distribution (Figure 5a), i.e., within the portion of body included in the *Operator volume*. The whiskers of the distribution reach 31 and 43 V/m. These values signify an attenuation ranging between 12 and 17 dB with respect to the unperturbed external E-field (Figure 3). Similar attenuation occurs at 35.5 MHz, as well as with antennas A3 and A4, with attenuations ranging between 17 and 22 dB. The highest attenuation is observed following exposure to Antenna 1, where it reaches 30 dB. Focusing the analysis on the head or expanding it to the whole body, the induced E-field values remain comparable, with variations that follow the local E-field distribution (Figures 6, 7). In Figures 6a, b Antenna 1 (25 MHz and 25 W of maximum operating power), and Antenna 2 (60.5 MHz and 50 W) are reported, respectively. Additional results for 16, 35.5, and 85.5 MHz are available in previous studies (28–30). Whereas the E-field distributions induced by Antennas 3 and 4 are shown in Figure 7. The maps are displayed using a color scale normalized to the 99.9^th^ percentile (E_99.9_) of the whole-body E-field relative to each antenna, so highlighting only local variations. The corresponding E_99.9_ values are summarized in Table 1. Antennas 1 (25 W, Figure 6a) and 2 (50 W, Figure 6b) produce similar upper-body field distributions, with hotspots near the clavicle (10.5 V/m for Antenna 1 and 74 V/m for Antenna 2) and the bend of the right arm (9 and 50 V/m, respectively). Particularly, the latter can be attributed to the close proximity of the arm with the metallic plane. As shown in Supplementary Figure S2, this configuration induces a capacitive effect that leads to a local accumulation of charges and a consequent increase in the electric field intensity, while the field in the surrounding space remains lower. On the neck and head, Antenna 2 generates a more symmetrical field distribution compared to Antenna 1. The lower exposure of the left arm by Antenna 1 (with field intensities < 1% of E_99.9_) is due to the position of the antenna which benefits less from the shielding effect produced by the manhole door. Considering the exposure of the lower limb, there is an evident difference between the effects of the two antennas: the vehicle act as a shield toward Antenna 1, justifying the lower intensities observed between Figures 5a–c. Conversely, at 60.5 MHz, the shielding effect of the vehicle is lost. An in-depth analysis on Antenna 2 (see Supplementary material for further details) showed that it induces a body response that closely resembles the behavior of a λ/2 dipole, as shown by the current distribution along the body in Supplementary Figure S1. This dipole-like behavior effectively reduces the shielding effect of both the manhole door and the vehicle structure, allowing the electromagnetic fields to concentrate at specific regions of the body. As a result, this phenomenon generates hotspots, particularly at the knees (50 V/m) and ankles (70 V/m), due to their reduced cross-section. At UHF frequencies (Figures 7a, b), the clavicle and right arm are the most exposed areas. Due to the Antennas' frequencies of work, these distributions are the most symmetrical, despite their different positions. Antenna 3 (50 W, Figure 7a) induces hotspots of 26 V/m in the neck and 20 V/m at the arm bend. The left arm shows uniform exposure with intensities ranging between 10 and 15 V/m. Antenna 4 (Figure 7b) is the only antenna generating its highest peak on the right arm (16.6 V/m), with intensities at the level of the clavicle up to 10 V/m. The lower limbs are shielded by the vehicle, justifying a halved whole-body median induced E-field, over the partial body one. The lower intensities in the internal organs are consistent with a reduced E field body penetration at UHF frequencies compared to HF and VHF. Overall, due to its positioning and operating frequency, Antenna 2 induces the highest coupling with the operator's body, resulting in the greatest induced E-field inside the body (Table 1). Figures 8, 9 present the SAR distributions corresponding to the E-field maps in Figures 6, 7, plotted on a logarithmic scale with 1 W/kg set to 0 dB. Despite external E-field hotspots, SAR levels remain below 1 W/kg in all scenarios. For Antenna 1 at 25 MHz, SAR patterns resemble previous findings at 16 MHz, with highest values below 50 mW/kg and located in the right arm and neck. Antenna 2, which produces the highest E-field intensities, also results in the highest SAR values, up to 0.7 W/kg at the arm bend and ankles. Antenna 3 induces symmetrical SAR distributions between the right and left sides of the body due to its central placement relative to the manhole. Upper-body SAR values remain below 10 mW/kg, while lower-body exposure is negligible (below 10^−5^ W/kg). Antenna 4 produces the highest SAR of 80 mW/kg at the right arm bend, and the lowest values across the trunk and other limbs (below 1 mW/kg). The lowest SAR values are observed in the thoracic organs. SAR results confirm compliance with ICNIRP guidelines, as summarized in Table 2. Whole-body average SAR (SAR_WB_) and peak localized SAR in the head (SAR_local, head_) remain well below the limits of 0.4 and 10 W/kg, respectively. For instance, SAR_WB_ is 0.00031 W/kg and SAR_local, head_ is 0.0092 W/kg for Antenna 1 at 16 MHz and 25 W. Similarly, at 85.5 MHz and 50 W (Antenna 2), SAR values remain two orders of magnitude below the limits (SAR_WB_: 0.06 W/kg; SAR_local, head_: 0.99 W/kg). Localized SAR in the limbs (SAR_local, limbs_) was also evaluated, with additional data provided in Table 3 and in previous studies (29, 30). Even in scenarios with external E-field hotspots near the arms, limb SAR values remain below the 20 W/kg limit. These findings emphasize that, while external E-fields may exceed ICNIRP reference levels, internal exposure remains minimal and fully compliant with ICNIRP safety standards.

**Table 1:** Maximum intensity of the E-Field induced inside the body.

	**Antenna 1, 25 MHz**	**Antenna 2, 60.5 MHz**	**Antenna 3**	**Antenna 4**
E_99.9_ (V/m)	10.5	74.5	26.3	16.6

**Table 2:** Evaluation of compliance with ICNIRP 2020 GUIDELINES: Whole body and head.

**Antenna**	**P_op, max_**	**Frequency**	SAR_Wb_ (W/kg)^*^	SAR_local, head_ (W/kg)^*^
1	25 W	16 MHz	0.00031	0.0092
		25 MHz	0.00088	0.019
2	50 W	35.5 MHz	0.0037	0.3
		60.5 MHz	0.05	0.6
		85.5 MHz	0.06	0.99
3	50 W	310 MHz	0.0093	0.19
4	20 W	351 MHz	0.0059	0.33
ICNIRP 2020 Limits			0.4	10

^*^SAR_Wb_, Whole-body average SAR; SAR_local, head_, peak of local SAR. SAR is averaged over a 10-g cubic mass in the head.

**Table 3:** Evaluation of compliance with ICNIRP 2020 GUIDELINES: Limbs.

	**ICNIRP 2020**	**Antenna #1, 25 MHz**		**Antenna #2, 60.5 MHz**		**Antenna #3**		**Antenna #4**	
		**Right**	**Left**	**Right**	**Left**	**Right**	**Left**	**Right**	**Left**
SAR_local, arm_(W/kg)^*^	20	0.022	0.83e-3	0.78	0.13	0.11	0.09	0.08	0.03
SAR_local, leg_ (W/kg)^*^	20	0.002	0.002	0.45	0.5	0.006	0.02	0.002	0.002

^*^SAR_local, limb_, Peak of local SAR. SAR is averaged over a 10-g cubic mass in the limbs (arms and legs).

## 4 Discussion

With the growing reliance on advanced communication systems and the proliferation of electromagnetic sources, it is increasingly important to ensure that human body exposure remains within international safety guidelines, that have distinct limits for general public and occupational exposure environments (10). EM exposure assessment of general public and workers in civilian scenarios has been extensively carried out in literature, both in far field and near field conditions, for frequencies spanning from HF up to the millimeter wave range (15–20, 34). Military contexts are generally inaccessible to civilians and are characterized by peculiar exposure conditions, where vehicular antennas are positioned near the operators. Hence a careful evaluation of exposure conditions is crucial to safeguard personnel from potential overexposure. Despite such unique challenges posed by military environments, studies addressing these scenarios remain limited. Furthermore, the lack of detailed descriptions of military exposure scenarios hampers accurate numerical modeling and precise assessment of compliance (23–27). Currently, the available literature on numerical studies in military contexts focuses on wearable systems, often evaluating their impact on performance rather than conducting comprehensive dosimetric assessments (23, 24). On the other hand, research on vehicle-mounted antennas does not consider realistic working conditions in terms of frequency, power and placement, and the dosimetric analyses performed are typically limited in scope (25–27). To address this gap and building on previous research (28–30), this study presents a comprehensive exposure assessment of a realistic military scenario, typical of the configurations most frequently encountered in operational practice. The evaluation focuses on analyzing the coupling between the electric fields generated by vehicular antennas and the human body, in a military context, where the near field condition is predominant. As these exposure scenarios are characterized by exceedance of the ICNIRP reference levels (RLs), it is crucial to perform the exposure assessment in the presence of the worker and to derive rule of thumbs on the compliance to the basic restrictions (BRs). Four antennas spanning the HF, VHF, and UHF bands were modeled. Specific frequencies and operational power were selected as provided by the CEPOLISPE to be representative of typical working conditions (6). As a first step, numerical simulations were carried out without including the human body model. As expected, the results showed that these antennas, accurately represented as simple monopoles due to their physical structure, generate electric field intensities that exceed the ICNIRP reference levels by up to five times in the near-field region, where the operator may be positioned. The extent of this phenomenon (Figure 3) depends on the antenna's characteristics, influenced by its frequency, input power and position with respect to the operator. As a consequence, these findings required further analysis incorporating a human body model, to investigate compliance with the ICNIRP basic restrictions. Dosimetric results obtained with the operator model showed different absorption patterns, nevertheless all induced values remained well below the ICNIRP BR, with an attenuation factor in terms of induced electric field ranging between 10 and 30 dB; this means at least a reduction of six times from the external E-field to the values induced inside the body. The operating frequency is the factor mostly driving the entity of the induced E field within the body. As shown in Figure 4, the distributions' frequency trend resembles the well-known RF absorption curves (35), with similar peaks in the range 60–80 MHz. At the operating frequency of Antenna 2, the attenuation rate between external and internal field is the lower among the antennas and ranges between 12 and 17 dB. Hence, Antenna 2 induces the highest induced E field at 60.5 and 85.5 MHz, consistent with findings from previous studies (28–30). This effect is due to the proximity of these frequencies to the human body's peak absorption spectrum (35–37). Additionally, at these frequencies, the body behaves as λ/2 dipole, and the shielding effect of the manhole and the vehicle almost negligible. A detailed description of this phenomenon is reported in the Supplementary material. For antennas operating in the HF range, the electric fields radiated in the free space can be as high as 130 V/m at 16 MHz (28) or 240 V/m at 25 MHz (for P_op, max_ = 25 W), however the absorption is very low, with an attenuation of 30 dB, hence the induced E-field remains below 10 V/m and the induced SAR values in the order of mW/kg. Such SAR values are comparable to those induced by Antenna 4 (351 MHz), which generated lower external E-field intensities below 100 V/m in the operator area for 20 W of P_op, max_. However, since the attenuation at UHF is lower (20 dB), signifying a higher absorption from the body, the induced E-field is comparable to that induced by Antenna 1. A notable case is Antenna 3, operating as well in the UHF range and with a P_op, max_ of 50 W. Due to its placement behind the manhole door, resulting in shielding effects, Antenna 3 induced a whole-body SAR comparable to Antenna 4, which operates in the same frequency range but with half the power. In all the evaluated conditions, a coupling effect between the body and the metallic plane of the vehicle is visible. This effect is particularly relevant for the right arm, that is bent and placed along the vehicle surface (Supplementary Figure S2A), and it is also visible on the left arm, where an increase of the E-field is visible in correspondence of the manhole extremities (Supplementary Figure S2B). Beyond localized electric field enhancements due to the proximity to metallic structures and the dipolar behavior of the body at VHF, the whole-body and localized SAR evaluations allow us to derive general considerations that can be extended to typical military exposure scenarios. Despite possible variability in antenna placement and operating conditions, being military applications standardized and given the spatial constraints of military vehicles, it is reasonable to assume that most exposure configurations will resemble those analyzed in this study, not only in the Italian national context but also in other countries. The simulations were conducted using the Duke male human model, which is representative of the demographic composition of military personnel assigned to tasks comparable to the simulated scenario (38). Given the differences in absorption between male and female models, where, in far-field conditions, males generally exhibit higher whole-body absorption peaks (17, 20–22), and considering that our results confirmed a similar frequency-dependent behavior at lower frequencies, the findings can be considered conservative when extended to female models. Future studies may nonetheless consider additional anatomical models to better account for inter-subject variability. Furthermore, this study is based on typical maximum operational powers, allowing a general rule of thumb to be outlined: antennas operating in the VHF band are expected to induce the highest levels of absorption in the body, HF antennas the lowest, and UHF antennas an intermediate level. In all cases, the typical placement on the vehicle, particularly with respect to structural elements such as the manhole, combined with the relatively low tissue conductivity at these frequencies (generally below 1 S/m), lead to SAR values that remained below ICNIRP basic restrictions. This result could not be predicted a priori, given that the external E-field values exceeded the guidelines' reference levels in all evaluated conditions. Therefore, this assessment provides generalizable insights that can support exposure evaluations in a broad range of realistic military scenarios. Beyond its technical contribution, the study offers practical guidance for safety management in operational contexts, particularly by demonstrating that Basic Restrictions (BR) are fulfilled even in configurations where Reference Levels (RL) are exceeded. These findings can assist military unit commanders in making informed decisions regarding personnel positioning during mission planning. Furthermore, the results may serve as a reference for regulatory bodies, contributing to the development of more tailored and context-specific exposure guidelines for military applications.

## 5 Conclusions

This paper provides a detailed and comprehensive analysis of various exposure scenarios, with a particular focus on military operators positioned with half their height outside the vehicle, a condition that represents one of the most significant exposure scenarios from an operational perspective. To the best of our knowledge, this is the first study to address these scenarios with such high degree of realism and detail in the evaluated conditions. The results indicate that while ICNIRP reference levels (RLs) are frequently exceeded, largely due to the near-field nature of the exposure and the power levels involved, compliance with basic restrictions (BRs) is consistently observed, even under conservative, worst-case conditions. The exposure patterns identified across the analyzed cases, together with the characteristic constraints and configurations of military vehicles, suggest that these findings could be extended to a wider range of comparable scenarios. Notably, a general trend was observed: VHF antennas tend to result in higher levels of absorption in the body, followed by UHF and then HF antennas. These insights contribute to the development of practical exposure guidelines relevant to real-world military operations, offering reassurance that, despite the common exceedance of RLs, adherence to BRs remains a key factor in supporting personnel safety. Nevertheless, given the sensitivity of exposure to factors such as antenna type and positioning, continued investigation is recommended to further refine predictive models and maintain effective exposure assessment in dynamic operational environments.

## Data Availability

The original contributions presented in the study are included in the article/Supplementary material, further inquiries can be directed to the corresponding author/s.
