# Supplementary material for: Assessing exposure from different vehicular antennas in military applications: a computational study
Source: Front Public Health. 2025 Aug 26;13:1620240. doi: 10.3389/fpubh.2025.1620240 (PMC12419222; doi:10.3389/fpubh.2025.1620240)
Supplement: Supplementary file 1 [file Supplementary_file_1.docx]

Supplementary Material

Assessing Exposure from Different Vehicular Antennas: a Computational study

Micol Colella^1^, Marianna Biscarini^1^, Daniele Ferrante^1,2^, Giovanni Pellegrino^2^, Marco De Meis^3^, Luca Mei^3^, Marta Cavagnaro^1^, Francesca Apollonio^1^, Micaela Liberti^1^

^1^Department of Information Engineering, Electronics and Telecommunications, Sapienza University of Rome, Rome, 00184, Italy

^2^Centro Polifunzionale di Sperimentazione (CEPOLISPE), Rome, Italy

^3^Larimart S.p.A., Rome, Italy

# Human body behavior at VHF frequency band

To evaluate the hypothesis that, at VHF frequencies and in particular at 60.5 MHz, the human body behaves as a λ/2 dipole, even in the near-field region, the body was sectioned at different heights to assess the current associated with each section. The current distribution, shown in Fig. S1, exhibits an almost perfectly cosinusoidal pattern, confirming the resonant dipole-like excitation of the human body. Notably, the portion of the body shielded by the manhole shows current levels comparable to those of the directly exposed part, consequently making the shielding effect of the manhole and the vehicle almost negligible. In Fig. S1(A), how the presence of the arm perturbs the current pattern is visible from the peak originating at the level of the bent arm. Removing the presence of the arm (Fig. S1(B)), consequently removes this peak. For all the antennas, a capacitive coupling between the arms and the metallic plane of the vehicle occurs. This effect is particularly relevant on the right arm, as it is bent along the plane. Nevertheless, it is present on the left arm as well. An example for Antenna S2 at 60.5 MHz is reported in Figure S2. The current distribution in Figure S2(A) is consistent with a low-impedance boundary condition at the level of the hand/forearm. The magnitude of the current entering the shoulder remains almost unchanged compared to the forearm. The peak in Figure S2(A) for the right arm, is consistent with the peak shown in Figure S1(A), whereas in Figure S2(B), the current trend for the left arm shows a progressive increase in current intensity as the length approaches the shoulder, maintaining values halved with respect to the right arm. This further confirms the coupling effect between the forearm and the metallic surface of the vehicle. The maps on the right illustrate the electric field map in the air and the induced field within the arm. The orientation and behavior of the field vectors confirm the influence of the proximity to the metallic plane, that perturbs the distribution, and deviates the vector directions, particularly in the outermost sections of the arm.


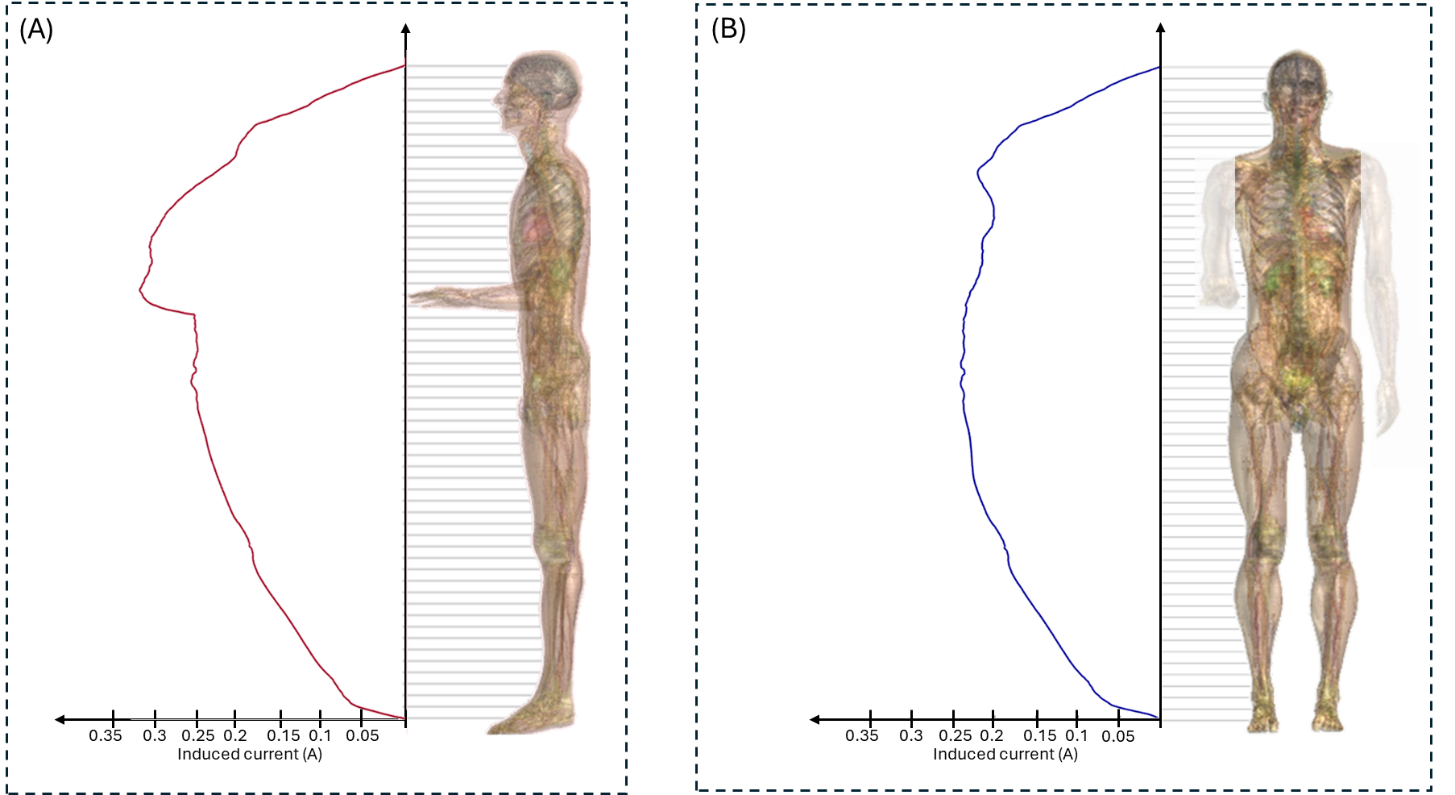


Supplementary Figure 1. Current distribution along the human body induced by Antenna S2 at 60.5 MHz, obtained by sectioning the body at different heights to evaluate the current associated with each section: (A) Behavior of the entire body. (B) Behavior of the entire body, excluding the arms.


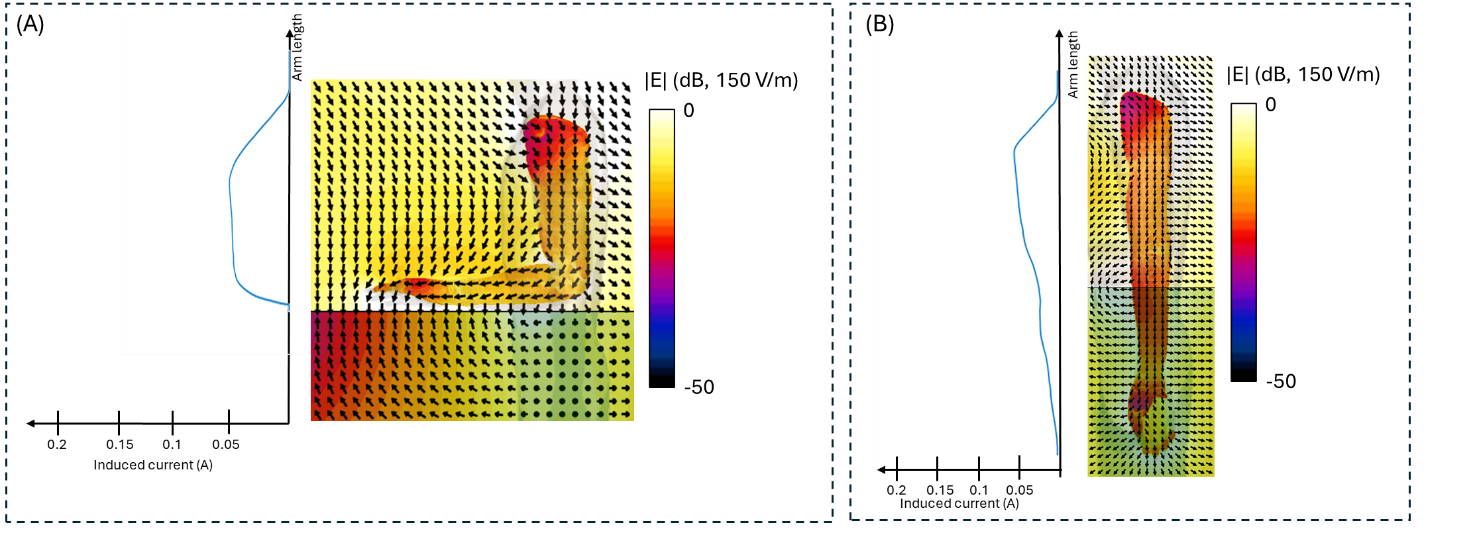


**Supplementary Figure 2.** Current along the length of the arm and E-field distribution for Antenna #2 at 60.5 MHz: (A) Right arm (bent along the vehicle), (B) Left arm (straight inside the vehicle). For each panel, on the left is the plot of the induced current, obtained following the same procedure for Supplementary Figure 1, on the right is the map of the E-field intensity in the space surrounding the arm and inside the arm. Superimposed is the vector field.
